# Non-inflammatory cerebral amyloid angiopathy as a cause of rapidly progressive dementia: A case study

**DOI:** 10.1590/S1980-57642009DN30400015

**Published:** 2009

**Authors:** Leonel Tadao Takada, Paulo Camiz, Lea T. Grinberg, Claudia da Costa Leite

**Affiliations:** 1 Cognitive and Behavioral Neurology Unit, Hospital das Clínicas da Faculdade de Medicina da Universidade de São Paulo, São Paulo SP, Brazil.; 2 Centro de Referência em Distúrbios Cognitivos (CEREDIC), Hospital das Clínicas da Faculdade de Medicina da Universidade de São Paulo, São Paulo SP, Brazil.; 3 Geriatrics Department, Hospital das Clínicas da Faculdade de Medicina da Universidade de São Paulo, São Paulo SP, Brazil.; 4 Memory and Aging Clinics, Department of Neurology, University of California, San Francisco.; 5 Pathology Department, Faculdade de Medicina da Universidade de São Paulo, São Paulo SP, Brazil.; 6 Radiology Department, Hospital das Clínicas da Faculdade de Medicina da Universidade de São Paulo, São Paulo SP, Brazil.

**Keywords:** dementia, cerebral amyloid angiopathy, vascular dementia

## Abstract

A 77 year-old men developed a subacute-onset, rapidly progressive cognitive decline. After 6 months of evolution, he scored 6 on the Mini-Mental State Examination and had left hemiparesis and hemineglect. The patient died 11 months after the onset of cognitive symptoms. Brain MRI showed microhemorrhages on gradient-echo sequence and confluent areas of white matter hyperintensities on T2-weighted images. Brain biopsy revealed amyloid-β peptide deposition in vessel walls, some of them surrounded by micro-bleeds. In this case report, we discuss the role of cerebral amyloid angiopathy (CAA) in cognitive decline, due to structural lesions associated with hemorrhages and infarcts, white matter lesions and co-morbidity of Alzheimer’s disease, as well as the most recently described amyloid angiopathy-related inflammation.

## Abstract (pt)

Um homem de setenta e sete anos desenvolveu quadro de início subagudo de demência rapidamente progressiva. Após seis meses de evolução, sua pontuação do Mini exame do estado mental foi de 6, e no exame neurológico apresentava heminegligência e hemiparesia esquerda. O paciente faleceu 11 meses após início dos sintomas. RM do encéfalo mostrou microhemorragias em sequência gradiente-echo e áreas confluentes de hiperintensidade de substância branca nas imagens pesadas em T2. Na biópsia cerebral observou-se deposição de peptídeo β-amilóide em vasos sanguíneos, alguns rodeados por microhemorragias. Neste relato de caso, discutimos o papel da angiopatia amiloide cerebral no declínio cognitivo, devido a lesões estruturais associadas a hemorragias e infartos, lesões de substância branca e comorbidade com doença de Alzheimer, assim como a mais recentemente descrita inflamação relacionada a angiopatia amiloide.

Rapidly progressive dementia (RPD), although more typically associated with prion diseases, can be caused by various conditions.^1^ These include neurodegenerative, autoimmune, toxic/metabolic and vascular etiologies, which must therefore be sought whenever investigating an RPD case. Here we present a case in which cerebral amyloid angiopathy (CAA) was found in a patient with RPD.

## Case report

A seventy-seven year-old, highly educated (23 years of schooling) man was admitted to the geriatrics ward of our hospital for a six-month cognitive decline investigation. In January, 2008, his wife noticed a subacute difficulty in signing checks and reading. After about a month, he started to neglect his own medications. Three months later, he started presenting memory deficits, with rapid progression (within a month he could not remember whether he had had lunch). By that point, he was also having delusions (such as saying his late father was home) and aggressive behavior. By May 2008, he developed urinary incontinence and later, gait difficulty (despite using right leg prosthesis for 4 years, his wife denied previous gait abnormalities). No hallucinations or seizures were reported. At the time of evaluation he was completely dependent for basic daily life activities.

He had suffered from diabetes mellitus and systemic arterial hypertension for thirty years. In his past medical history, he had also undergone a right hip replacement and right transtibial amputation (for which he used a prosthesis for ambulation). He had suffered a head trauma with loss of consciousness at the age of 12. No previous history of stroke was reported.

No family history of dementia was reported.

Upon neurological examination, he had hemineglect and anosognosia. His language skills appeared preserved, though he tended to confabulate and was dysarthric. His mini-mental state examination^2^ score was 6 (out of 30), having scored two points on spatial orientation, two points on immediate recall, one on naming and one on repetition. Semantic verbal fluency (animals) was zero. Bilateral ideomotor apraxia and optic ataxia were also seen. He was able to stand only with bilateral support and had left hemiparesis with deep tendon reflex asymmetry.

Blood testing revealed iron deficiency anemia (hemoglobin 10.8 g/dL and hematocrit 33.9%) and fasting blood glucose level of 202 mg/dL with a glycosylated hemoglobin level of 9.6%. Anti-peroxidase and anti-tireoglobulin antibodies were below detection levels. Other biochemical tests, as well as vitamin B12 and folic acid levels, were normal. HIV and hepatitis B and C serology were negative. Erythrocyte sedimentation rate (ESR) was normal. Anti-neutrophil cytoplasmic antibody (ANCA), glutamic acid decarboxylase (GAD) antibodies, rheumatoid factor, anti-SS antibodies, serum protein electrophoresis, were all normal (or negative). Anti-nucleus antibodies (ANA) were detected in 1:80 titers.

Spinal fluid analysis showed normal cellularity and increased protein levels (60 mg/dL), with slightly high gammaglobulin levels (14.9%). Opening pressure was normal (9 cmH_2_O). *Treponema pallidum* immunology was negative (both in serum and spinal fluid).

Electroencephalogram showed diffuse disorganization of brain electrical activity and left temporal acute wave paroxysms. Gastrointestinal endoscopy revealed an active gastric ulcer, which was treated with proton pump inhibitor. Thoracic and abdominal computed tomography revealed no abnormal masses.

Neuroimaging findings are described below. During investigation, the spinal tap test was performed twice, but no cognitive or gait improvement was observed. Due to the rapidly progressive character of evolution, a right frontal cerebral biopsy was performed (also described below).

### Neuroimaging findings

A MRI was performed on a 1.5 Tesla MR unit. The MRI protocol included: Axial diffusion-weighted image (TR=8000 ms, TE=97 ms, b=0 and b=1000, and slice thickness=5 mm), axial T2* (TR=450 ms, TE=15 ms, flip angle 20o and slice thickness=5 mm), axial unenhanced T1-weighted image (TR=400 ms, TE=11 ms, and slice thickness=5mm), axial FLAIR (fluid attenuated inversion recovery) image (TR=11002 ms, TE=103 ms, TI=2400 ms, and slice thickness=5 mm), coronal T2-weighted fast spin echo TR=4730 ms, TE=99ms, and slice thickness=5 mm), and axial enhanced T1-weighted image (TR=400 ms, TE=11 ms, and slice thickness=5 mm) and axial volumetric spoiled gradient recalled echo (SPGR) (TR=6.6 ms, TR=2.0, slice thickness=1.8 mm, and interspace=0.8 mm) after gadolinium administration.

**Figure 1:**
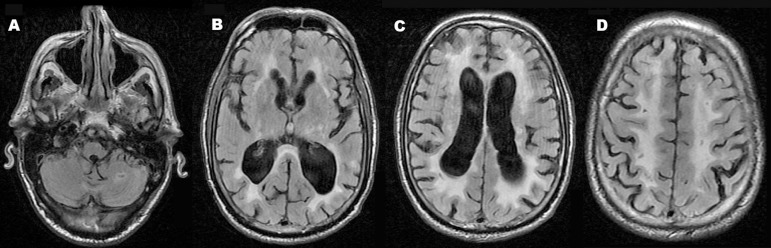
Axial FLAIR images. Note a hyperintense lesion with a hypointense core in the left cerebellar hemisphere [A]. There is ventricular dilatation and sulcal enlargement with multiple hyperintense lesions in the basal ganglia, thalamus, internal and external capsules and in the periventricular white matter [B-C]. There are also lesions in the subcortical area [D].

**Figure 2:**
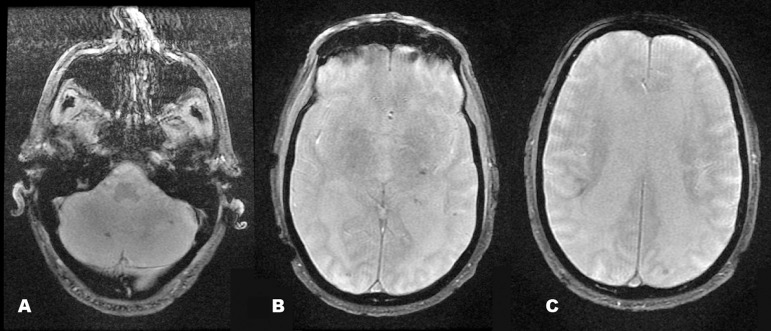
Axial T2* images showing multiple areas with marked hypointensity suggestive of hemossiderin content [A-C].

The MRI (Figure 1) showed ventricular dilatation and sulcal enlargement greater than the expected for the patient’s age. The patient presented multiple hyperintense lesions in the cerebellum, pons, mesencephalon, basal ganglia, internal and external capsules, thalamus and periventricular, and subcortical white matter. These lesions may have represented microleukoangiopathy and/or gliosis. The T2* (Figure 2) images showed multiple marked hypointense foci suggestive of hemossiderin. The enhanced images showed no contrast enhancement. Computed tomography of the head demonstrated no calcified lesions.

### Neuropathological examination

Three fragments of frontal cortex, the biggest measuring 0.7cm, were removed via stereotactic brain biopsy.

**Figure 3:**
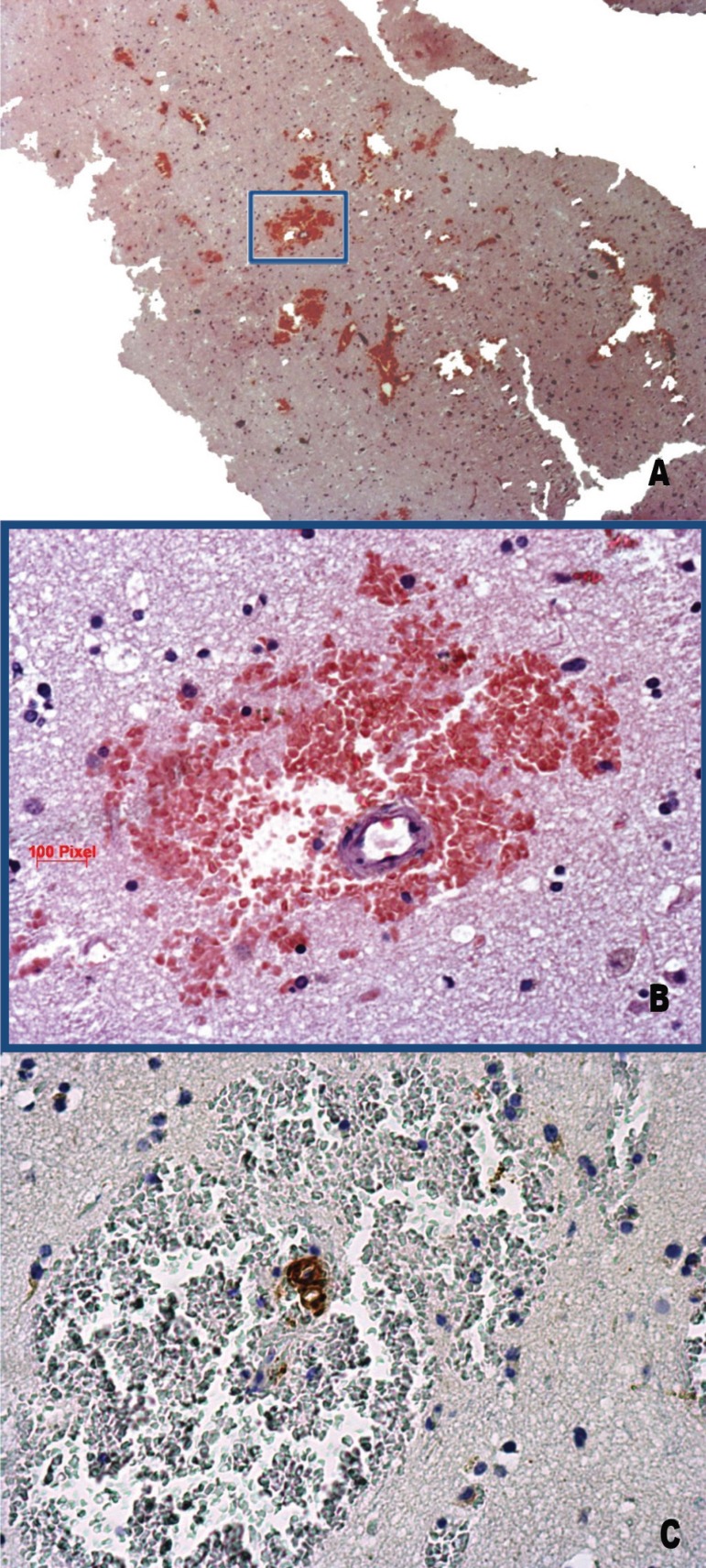
[A] frontal cortex depicting multiple foci of micro-bleeding. HE. 25x. [B] Higher magnification of the boxed area in 3A. Note that the micro-bleeding is surrounding a vessel in which the wall is thickened by the deposition of amorphous material. 200x. [C] immunostaining against Amyloid-β. The brownish color of the vessel indicates positivity for Amyloid-β protein. 400x

The small vessels of the gray matter were thickened by the deposition of amorphous material as detected using routine hematoxylin-eosin (HE) staining. Some of these vessels were surrounded by acute and semi-acute micro-bleedings (Figures 3A and 3B).

Furthermore, the tissue was immunostained against: monoclonal mouse anti-Amyloid-β 1:5000 (4G8, Covance, Emeryville, CA), monoclonal mouse anti-phospho-tau 1:1000 (PHF-1, gift of Peter Davies, New York, NY) and polyclonal rabbit anti-alpha-synuclein 1:10000 (EQV-1, gift of Kenji Ueda, Tokyo, Japan).

The anti-Amyloid-β immunostaining revealed deposition of Amyloid-β protein in the vessel walls of the cerebral gray matter corresponding to the amorphous material deposition observed on HE staining (Figure 3C). Scattered amyloid diffuse plaques were also seen in the neuropil. Neuritic plaques, dystrophic neuritis and neurofibrillary tangles (NFT) and Lewy bodies were not seen in tissue.

The patient was diagnosed as having cerebral amyloid angiopathy with micro-bleedings. Due to the scarcity of available tissue, it was not possible to rule out Alzheimer’s disease, despite the fact that neuritic plaques and neurofibrillary tangles were not found.

### Evolution

After his discharge from hospital, the patient could no longer walk (even with aid), and developed pressure ulcers. His cognitive functions deteriorated rapidly and he died due to pneumonia in December, 2008, eleven months after the onset of symptoms.

## Discussion

We presented the case of a 77 year-old patient with rapidly progressive dementia (RPD). Our initial diagnostic hypotheses included prion disease or autoimmune encephalopathy, but ancillary tests were not supportive. At this point, because of the rapid rate of cognitive decline, a brain biopsy was ordered which confirmed a diagnosis of cerebral amyloid angiopathy (CAA).

CAA is characterized by the deposition of amyloid proteins within leptomeningeal and cortical vessels, with the β-amyloid type being the most common^3,4^. CAA may manifest clinically not only as lobar hemorrhage – the most recognized manifestation – but also as ischemic infarctions, subarachnoid hemorrhage, transient focal neurological manifestations (caused by focal seizures or transient ischemic attacks) or cognitive decline^3,5^.

CAA has been linked to aging, dementia and Alzheimer’s disease (AD). It is known that the prevalence of CAA neuropathological findings increase with advancing age^4,6^. CAA is also a recognized cause of cognitive impairment^7^, and was associated with a higher prevalence of dementia in a neuropathological study^8^.

MRI findings in CAA include cortico-subcortical microhemorrhages or petecchial hemorrhages (seen in gradient-echo sequences)^9,10^ and white matter hyperintensities^4^. Microbleeds on gradient-echo T2-weighted MRI have been associated with cognitive impairment, particularly executive impairment in nondemented individuals^11,12^. Although gradient-echo MRI is considered a sensitive test for CAA (by revealing hemorrhagic lesions)^10^, in this case, a discrepancy between neuroimaging findings with a relative paucity of hypointense lesions in MRI T2*-weighted images and neuropathological findings of multiple microbleeds was noted. There is also the possibility that acute-appearance microbleeds may have occurred during the biopsy procedure which might explain the disparity.

White matter lesions in CAA are believed to be caused by chronic hypoperfusion of the deep white matter associated with vascular changes of long perforating arteries^4^. It is relevant, however, that despite the fact that white matter abnormalities are characteristic in CAA, severe and diffuse white matter hyperintensities (resembling subcortical arteriosclerotic encephalopathy) such as those seen in this case, are considered rare by some^13^.

CAA has also been associated with AD, after the frequent observation of CAA in AD patients (between 78 and 100%^14,15^), and because patients with AD and CAA have showed more widespread distribution than non-demented individuals with CAA^6^. Capillary occlusion or narrowing caused by CAA may explain the hypoperfusion that is believed to occur in the AD brain^6^ and thus may be a part of its pathophysiology^16,17^.

More recently, CAA and RPD have been linked with a form of inflammatory vasculopathy, known as cerebral amyloid inflammatory vasculopathy^18^ or amyloid angiopathy-related inflammation^19,20^. For reasons still unknown, a subset of patients with severe CAA may develop a picture of encephalopathy with subacute cognitive decline, seizures and/or headache that may respond (with both clinical and radiological improvement) to anti-inflammatory treatment^19^. Brain MRI reveals large and confluent areas of white matter hyperintensities in T2/FLAIR-weighted images and scattered microbleeds on gradient-echo sequences^19^. Neuropathological evaluation shows perivascular inflammation with mononuclear and multinucleated white blood cells surrounding vessels stained positive for Aβ (which raised the possibility of Aβ being the trigger to immune response) as well as multiple microbleeds and small infarctions in the cerebral cortex besides rarefaction of the underlying white matter^20^. Although the patient did not receive immunosuppressive therapy, this form of inflammatory vasculopathy cannot be considered the diagnosis, as no inflammatory signs were found in the cerebral biopsy.

One may question whether CAA was the only cause of RPD in this patient. Indeed, one of the limitations of this report is the lack of sufficient neuropathological data to either confirm or exclude the diagnosis of AD. Only diffuse plaques were observed, and thus neither Braak & Braak stage (based on the finding of neurofibrillary tangles)^21^ or Consortium to Establish a Registry for Alzheimer’s Disease (CERAD) criteria (based on neuritic plaques)^22^ for AD were fulfilled. However, the brain fragment analyzed consisted of biopsy specimen and thus the co-morbidity of AD could not be ruled out. In contrast with CAA, which is more commonly and precociously found in the neocortex^6^, neuritic plaques and neurofibrillary tangles are observed in the isocortex in more advanced phases of AD^21^ and a vascular amyloid finding is not directly correlated with amyloid plaque deposition^23^. The sensitivity of brain biopsy for the diagnosis of dementia should also be considered. As reviewed by Warren et al.^24^, across 17 studies, a specific diagnosis was achieved in 22-84% of brain biopsies. In a series of patients with rapidly progressive neurological conditions, Josephson et al.^25^ found that brain biopsies had a sensitivity of 65% for the diagnosis (the most common being lymphoma and Creutzfeldt-Jakob disease). A positive ANA was found in low titers (1:80). However, this patient presented no other signs of systemic autoimmune disorder and a false positive ANA may be found in low serum dilutions in controls (with 1:160 being suggested as a cutoff)^26^.

On one hand, there are reports of CAA solely causing RPD^5^ while vascular dementia is known to be a cause of RPD^1^. On the other hand, CAA is known to lower the threshold for dementia caused by neurodegenerative conditions (such as AD) and RPD is usually associated in the context of CAA with amyloid angiopathy-related inflammation 3 – which as stated above was not the case in this patient. The patient’s clinical co-morbidities, such as anemia and poorly compensated diabetes (as shown by high glycosylated hemoglobin) could have also hastened the cognitive decline. Either way, irrespective of whether CAA was the sole cause of RPD in this patient or not, we believe this report serves to raise the awareness of CAA as a cause of RPD in patients with leukoencephalopathy and microhemorrhages.
